# Molecular analysis of lipoid proteinosis: identification of a novel nonsense mutation in the *ECM1 *gene in a Pakistani family

**DOI:** 10.1186/1746-1596-6-69

**Published:** 2011-07-26

**Authors:** Muhammad Nasir, Amir Latif, Muhammad Ajmal, Reem Qamar, Muhammad Naeem, Abdul Hameed

**Affiliations:** 1 Institute of Biomedical and Genetic Engineering, GPO Box 2891, 24-Mauve Area, G-9⁄1, Islamabad, Pakistan; 2 Leprosy Hospital, Rawalpindi, Pakistan; 3 Department of Biotechnology, Quaid-i-Azam University, Islamabad-45320, Pakistan

**Keywords:** *ECM1*, genodermatoses, mutation, Pakistan

## Abstract

Lipoid proteinosis is a rare autosomal recessive disease characterized by cutaneous and mucosal lesions and hoarseness appearing in early childhood that is caused by homozygous or compound heterozygous mutations in the *ECM1 *gene located on chromosome 1q21. The aim of the study was to investigate the molecular genetic defect underlying lipoid proteinosis in a consanguineous Pakistani family.

**Methods:**

Genotyping of seven members of the family was performed by amplifying microsatellite markers, tightly linked to the *ECM1 *gene. To screen for mutations in the *ECM1 *gene, all of its exons and splice junctions were PCR amplified from genomic DNA and analyzed by SSCP and sequenced directly in an ABI 3130 genetic analyzer.

**Results:**

The results revealed linkage of the LP family to the *ECM1 *locus. Sequence analysis of the coding exons and splice junctions of the *ECM1 *gene revealed a novel homozygous mutation (c.616C > T) in exon 6, predicted to replace glutamine with stop codon (p.Q206X) at amino acid position 206.

**Conclusions:**

The finding of a novel mutation in Pakistani family extends the body of evidence that supports the importance of *ECM1 *gene for the development of lipoid proteinosis.

## Background

Lipoid proteinosis (LP; MIM 247100) also known as Urbach-Wiethe disease or hyalinosis cutis et mucosae, was first reported by Urbach and Wiethe, in 1929 [1]. It is a rare genetic disease, which is inherited in an autosomal recessive fashion. The disease occurs worldwide but is more common in certain geographical areas such as the Northern Cape province of South Africa, including Namaqualand. Clinical heterogeneity is reported in LP [2], although it usually presents in early childhood with hoarseness, caused by infiltration of the laryngeal mucosa [3]. Skin lesions or pox-like scars usually appear simultaneously or shortly afterwards. Other characteristic findings include the arrangement of 'beaded' waxy papules, known as moniliform blepharosis, which may be present along the margins of both eyelids [4,5]. Histological and ultra structural examination has revealed the widespread deposition of hyaline-like material and disruption/reduplication of basement membrane around blood vessels and at the dermal-epidermal junction, mouth and upper respiratory tract, and other internal organs [2,6]. Overproduction of normally expressed non-collagenous protein in the hyaline material has also been reported [7]. Tongue is often firm and its mobility may be limited. Other symptoms may include thickening of frenulum, scarring, warty skin papules, nail dystrophy, dental anomalies and some neuropsychiatric symptoms [8,9]. Molecular genetic studies of LP linked the disorder to chromosome 1q21.1 [2]. The responsible gene was identified as *ECM1*, which encodes for the glycoprotein extracellular matrix protein 1. To date, several mutations in the *ECM1 *gene have been reported in unrelated LP families from different geographical areas. In this study we report a novel nonsense mutation in a consanguineous Pakistani family affected with lipoid proteinosis; and an update of *ECM1 *gene mutation data base.

## Methods

### Subjects

**Figure 1:**
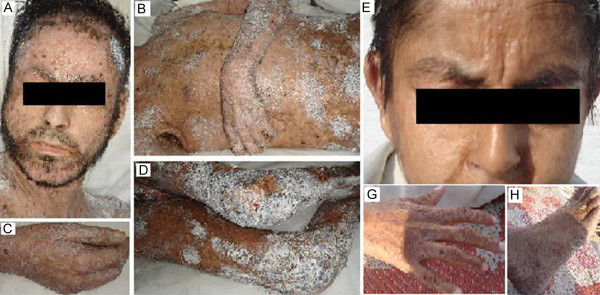
**Clinical presentation of the lipoid proteinosis patients**. (A) A 23 years old patient with yellow-white infiltrates and diffuse acneiform scars on the face; yellow discolouration of lips is also prominent; (B) Infiltrated skin of the trunk and upper extremities with numerous scars; (C, D, G, and H) Warty skin, thickening & infiltration on the hands, legs, knees and foot. (E) A 15 years old patient with nodular or diffuse yellow waxy infiltrates located on the face; pseudo solar elastosis of the cheeks and forehead.

A consanguineous Pakistani family with autosomal recessive LP was ascertained from Rawalpindi district. Two individuals (ages 15 and 23 years) in the family were affected with the disorder (Figure 1). Detailed clinical examination of all the family members, including affected individuals (IV-1, IV-2), their parents (III-1, III-2) and normal siblings (IV-3, IV-4, IV-5), was carried out by a dermatologist at local Leprosy Hospital. Before the start of the study, approval was obtained from the Quaid-i-Azam University institutional review board. In addition, informed consent was obtained from the family members who participated in the study. Blood samples from patients, their parents and 3 unaffected siblings were collected and genomic DNA was extracted by the standard phenol-chloroform method. DNA samples from 100 ethnically matched unrelated normal Pakistani individuals were also collected as controls.

### Genotyping

Genomic DNA from seven individuals of the family was genotyped using microsatellite markers tightly linked to the *ECM1 *gene. Polymorphic microsatellite markers were polymerase chain reaction (PCR) amplified. Each reaction was carried out in 10 μl volume containing 1.5 mM MgCl2, 0.6 μM of each primer, 0.2 mM each dNTPs, 1U *Taq *DNA polymerase and 1 × PCR buffer {16 mM (NH4) 2SO4, 67mMTris-HCI (pH 8.8), and 0.01% of the nonionic detergent Tween-20} (Bio-line, London, UK). Amplification was performed with an initial denaturation for 5 min at 94°C, followed by 35 cycles of denaturation at 94°C for 45 sec, annealing at 55°C for 45 sec, extension at 72°C for 45 sec and a final extension at 72°C for 10 min. The PCR products were separated on 8% non-denaturing polyacrylamide gels stained with ethidium bromide and alleles were assigned by visual inspection.

### Mutation analysis

For detection of mutation in the *ECM1 *gene, 8 sets of primers were used to amplify all coding exons and adjacent splice sites by PCR. PCR products were initially screened for mutations by single stranded conformational polymorphism (SSCP) analysis. For this, aliquots of 10 μl of each PCR product was mixed with 10 μl denaturing solution (95% formamide, 20 mM EDTA pH 8.0, 0.05% xylene-cyanole and 0.05% bromo phenol blue), heated for 7 min at 95°C in PxE thermal cycler (Hybaid, Basingstoke, U.K.) and chilled quickly on ice for 5 min. Denatured DNA was subjected to 8% polyacrylamide gel electrophoresis (20 × 20 × 0.1 cm) containing 7% glycerol and 1 × tris-borate EDTA (TBE) buffer at constant 30W for 3.5-4.0 hrs. The gels were stained with ethidium bromide (1 μg/ml) in 1 × TBE buffer for about 5 min and visualized under UV transilluminator gel documentation system (Syngene, UK). The PCR products with mobility shift were then purified for DNA sequencing using commercially available QIAquick PCR Purification Kit (Qiagen, Crawley, U.K.). Direct sequencing was carried out by using Big Dye^® ^Terminator v3.1 cycle sequencing kit in an ABI 3130 genetic analyzer (Applied Biosystems, Foster City, CA, U.S.A.).

## Results

### Clinical details

The affected individuals had hoarseness of voice, pseudo-solar elastosis of the cheeks and forehead and waxy papules along the margins of eyelids. Progressive thickening and scarring of the skin and mucous membranes, hyperkeratosis with warty papules on the palms and dorsum of the hands, elbow and knee were also observed. Mobility of the tongue was limited with yellow discoloration of the lips. The clinical features of LP were identical in both patients; however, severity was varied, which may be due to difference in age as many of the clinical features of LP only manifest fully with time. Both the affected individuals showed initial symptoms during infancy. The heterozygous parents and siblings revealed no clinical signs and symptoms of LP upon detailed skin examination.

### Genotyping

**Figure 2:**
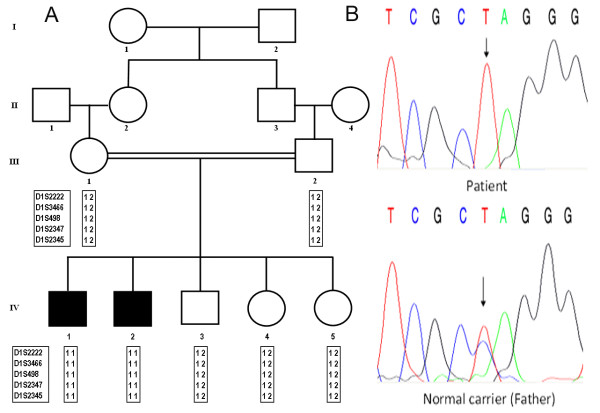
**The LP family pedigree and DNA sequence analysis**. (A) A multigenerational consanguineous Pakistani family in which lipoid proteinosis (LP) is segregating as an autosomal recessive trait. (B) The electropherogram of an affected member revealed a homozygous C > T transition at nucleotide position c.616 in exon 6 while father's electropherogram revealed heterozygosity at the same position.

Genotyping of two affected and five normal individuals of the family (Figure 2A) was performed with microsatellite markers (D1S2222, D1S3466, D1S498, D1S2347, D1S2345) mapped in the region of *ECM1 *gene. The markers were fully informative, and the results revealed that the affected individuals were homozygous for the markers suggesting linkage to the *ECM1 *gene.

### Mutation analysis

The SSCP analysis revealed mobility shift bands in PCR products of exon 6 of the *ECM1 *gene. Mutant allele was found to be co-segregating with the disease phenotype in the family. Both affected individuals (IV-1, IV-2) in the family were homozygous for the mutant allele, while their parents (III-1, III-2) and normal siblings (IV-3, IV-4, IV-5) were heterozygous.

Direct sequencing of PCR products amplified from the affected individuals revealed a homozygous C > T transition at nucleotide position 616 (c.616C > T) in exon 6 of the *ECM1 *gene (Figure 2C). To see the effect of substitution on the coding sequence, the nucleotide sequence was analyzed with CLC Workbench 4.0.2 software. The C > T transition at nucleotide 616 changes the codon for glutamine at amino acid position 206 into a stop codon (p.Q206X), predicting premature termination of ECM1 with 205 amino acids instead of 540 amino acids of the normal ECM1 protein. The mutation was confirmed by bidirectional DNA sequencing. The parents and three normal individuals of the family were heterozygous for the mutation. To ensure that the mutation does not represent a neutral polymorphism in the Pakistani population, 100 ethnically matched healthy control individuals were screened for the mutation by PCR followed by direct sequencing. The mutation was not identified outside the family.

## Discussion

The *ECM1 *gene comprises of 10 exons and encodes for the extracellular matrix protein 1. There are four splice variants including ECM1a, ECM1b and ECM1c, encoding proteins of 540, 415 and 559 amino acids, respectively. The recently described fourth splice variant comprises transcription of 71 bp at the 3' end of intron 1 and part of exon 2 to give a truncated 57 amino acid protein [10]. ECM1 is a negative regulator of endochondral bone formation, inhibiting alkaline phosphatase activity and mineralization [11]. It promotes angiogenesis, and shows over expression in certain malignancies. It interacts with a variety of extracellular and structural proteins, contributing to the maintenance of skin integrity and homeostasis [12-14]. The expression studies have demonstrated presence of ECM1a within basal keratinocytes and ECM1b in suprabasal cells, suggesting a role for ECM1 in terminal keratinocyte differentiation [15].

Forty six mutations in the *ECM1 *gene have been described so far in unrelated patients affected with lipoid proteinosis (Table 1). These include 19 insertions/deletions, 15 nonsense, 8 missense and 4 splice site mutations. Both homozygous and compound heterozygous genotypes have been reported. About half of all mutations (22 of 46) are located within exon 6 or 7 (including adjacent splice sites). Therefore, sequencing of these two exons has been suggested as the initial step in efficiently determining the molecular pathology in new cases of LP. Furthermore, as previously reviewed by Chan et al 2007, there is no clear paradigm for genotype phenotype correlation, considering the type and position of the *ECM1 *gene mutations reported so far. Most of the mutations in the *ECM1 *gene are specific to individual families and only few are recurrent. In the present study, we identified a novel nonsense mutation (Q206X) in exon 6 of the *ECM1 *gene in a consanguineous Pakistani family. Five ECM1 mutations have been previously reported in unrelated Pakistani LP families (Table 1) and to our knowledge there is no report of recurrent mutation. The Q206X mutation is predicted to result in the increased degradation of both full-length ECM1a and ECM1b transcripts due to nonsense mediated mRNA decay mechanism. Exceptionally, ECM1 transcripts might be expressed stably leading to production of truncated ECM1 protein. Although, functional consequences of the premature termination codon mutations must be established by northern blotting or quantitative reverse transcriptase PCR, previous reports have not suggested any difference in phenotype related to different mutation genotypes. Premature stop codons in the last exon lead to the presence of ECM1 truncated protein because the non sense mediated mRNA decay is dependent on an upstream exon-exon junction [16]. However, such patients did not show a different or milder phenotype in comparison with cases with more upstream mutations [17,18].

**Table 1:** Summary of the mutations in the *ECM1 *gene reported so far

Position	Sequence change	Mutation type	Predicted protein change	Patient origin	Reference
Genotype: homozygous					
Intron 1	IVS1+1G>C	Splice site	Removal of the translation initiation site	Israeli Arab, Kuwaiti, Egyptian	10, 17
Exon 2	c.93G>T	Missense	p.R31S	Libyan	19
Exon 2	c.94 C>T	Nonsense	p.Q32X	Libyan, Indian	17, 19
Exon 3	c.157 C>T	Nonsense	p.R53X	Japanese	20, 21
Exon 3	c.220 C>T	Nonsense	p.Q74X	Indian	17
Exon 4	c.243delG	Deletion	In-frame deletion of 61 amino acids	Thai	20
Exon 5	c.340 C>T	Nonsense	p.Q114X	Japanese	17
Exon 6	c.499 T>C	Missense	p.F167L	Polish	22
Exon 6	c.501insC	Insertion	Frame shift	Dutch, Belgian	2,23
Exon 6	c.507delT	Deletion	Frame shift	Japanese, Thai, Indian, Canadian, Iranian, Turkish, Pakistani, Chinese	17,20,23,24, 25,26
Exon 6	c.541del3ins16	Indel	Frame shift	Brazilian	27
Exon 6	c.589 C>T	Nonsense	p.Q197X	Italian	28
Exon 6	c.629 T>C	Missense	p.L210P	French	17
Exon 6	c.658 T>G	Missense	p.C220G	Chinese	29, 30
Exon 7	c.727 C>T	Nonsense	p.R243X	Belgian	31
Exon 7	c.735delTG	Deletion	Frame shift	Turkish	20
Exon 7	c.742 G>T	Nonsense	p.E248X	Indian	32
Exon 7	c.785delA	Deletion	Frame shift	Indian	20
Exon 7	c.806 G>A	Missense	p.C269T	Saudi Arabian	33
Exon 7	c.826 C>T	Nonsense	p.Q276X	South African	2
Exon 7	c.892delC	Deletion	Frame shift	Japanese	20
Exon 7	c.1036 C>T	Nonsense	p.Q346X	Pakistani	2
Exon 7	c.1077 G>A	Nonsense	p.W359X	British	2, 17
Exon 7	c.1019delA	Deletion	Frame shift	Kuwaiti	2
Intron 7	IVS7+1G>A	Splice site		Pakistani	17
Exon 8	c.1106 A>G	Missense	p.H369C	Indian	17
Exon 8	c.1190insC	Insertion	Frame shift	American	20
Exon 8	c.1209ins62	Insertion	Frame shift	Pakistani	34
Exon 8	c.1246 C>T	Nonsense	p.R416X	Indian	17
Exon 8	c.1253delC	Deletion	Frame shift	British	17
Exon 8	c.1300delAA	Deletion	Frame shift	Saudi Arabian	17, 33
Intron 8	IVS8+1G>A	Splice site		Pakistani	17
Intron 8 - Intron 10	IVS8_IVS10del	Deletion	Deleterious effect on protein structure and function	Saudi Arabian	2, 33
Exon 10	c.1393delA	Deletion	Frame shift	Israeli	18
Exon 10	c.1426 C>T	Nonsense	p.R476X	Indian	17
Exon 10	c.1441 C>T	Nonsense	p.R481X	Indian	17
Genotype: compound heterozygous					
Exon 1	c.29 T>G	Missense	p.V10G	Polish	17
Intron 1	IVS1+1G>A	Splice site			
Exon 3	c. 157 C>T	Nonsense	p.R53X	Spanish	17
Exon 6	c.603delTG	Deletion	Frame shift		
Exon 4	c.240delTC	Deletion	Frame shift	German	18
Exon 7	c.1019delA	Deletion	Frame shift		
Exon 4	c.283 C>T	Nonsense	p.Q95X	British	17
Exon 10	c.1432delA	Deletion	Frame shift		
Exon 6	c.480 G>A	Nonsense	p.W160X	Canadian	20
Exon 6	c. 499 T>A	Missense	p.F167I		
Exon 6	c.542insAA	Insertion	Frame shift	Italian	20
Exon 7	c.727 C>T	Nonsense	p.R243X		
Exon 6	c.543delTG/ins15	Indel	Frame shift	Italian	35
Exon 7	c.727 C>T	Nonsense	p.R243X		
Exon 6	c.658 T>G	Missense	p.C220G	Chinese	36
Exon 10	c.1426 C>T	Nonsense	p.R476X		
Exon 7	c.727 C>T	Nonsense	p.R243X	Italian	17
Exon 7	c.735delTG	Deletion	Frame shift		

## Conclusions

We have identified a novel nonsense mutation in exon 6 of the *ECM1 *gene in a Pakistani family extending the mutation spectrum of the gene. The study extends the body of evidence that supports the role of *ECM*1 gene in the development of lipoid proteinosis. Identification of pathogenic mutations in the *ECM1 *gene should be helpful to improve genetic counseling and DNA based prenatal diagnosis.

## Competing interests

The authors declare that they have no competing interests.

## Authors' contributions

MN^1 ^performed experimental work, AL performed clinical study of the family, MA participated in experimental work, RQ updated mutation database and participated in manuscript preparation, MN^2 ^analyzed data and prepared manuscript, AH designed research plan and analyzed data. All authors read and approved the final manuscript.
